# Acute Febrile Illness Caused by *Brucella Abortus* Infection in Humans in Pakistan

**DOI:** 10.3390/ijerph16214071

**Published:** 2019-10-23

**Authors:** Arbab Saddique, Shahzad Ali, Shamim Akhter, Iahtasham Khan, Heinrich Neubauer, Falk Melzer, Aman Ullah Khan, Asima Azam, Hosny El-Adawy

**Affiliations:** 1 Animal Physiology Laboratory, Department of Zoology, Pir Mehr Ali Shah Arid Agriculture University, 46000 Rawalpindi, Pakistan; saddiquearbab@gmail.com (A.S.); sashraf1993@gmail.com (S.A.); 2 Wildlife Epidemiology and Molecular Microbiology Laboratory (One Health Research Group), Discipline of Zoology, Department of Wildlife and Ecology, University of Veterinary and Animal Sciences, Lahore, Ravi Campus, 55300 Pattoki, Pakistan; 3 Section of Epidemiology and Public Health, University of Veterinary and Animal Sciences, Lahore Sub- campus, 35200 Jhang, Pakistan; drkhan_uaf@yahoo.com; 4 Friedrich-Loeffler-Institut, Institute of Bacterial Infections and Zoonoses, Naumburger Str. 10 96a, D-07743 Jena, Germany; heinrich.neubauer@fli.de (H.N.); falk.melzer@fli.de (F.M.); AmanUllah.Khan@fli.de (A.U.K.); 5 Department of Pathobiology, College of Veterinary and Animal Sciences, 35200 Jhang, Pakistan; 6 Department of Zoology, Shaheed Benazir Bhutto Women University, 25000 Peshawar, Pakistan; asimaazam786@gmail.com; 7 Faculty Medicine of Veterinary, Kafrelsheikh University, 33516 Kafr El-Sheikh, Egypt

**Keywords:** human brucellosis, RBPT, ELISA, risk factors, real-time PCR

## Abstract

Brucellosis is a zoonosis of great and worldwide public health concern that can cause a severe febrile illness in humans. In Pakistan, brucellosis is a critical problem in both animals and humans. This study aimed to gain insight into its prevalence and to analyze the potential risk factors of patients with acute febrile illness (AFI) of an unknown cause, at the hospitals of Rawalpindi and Islamabad in Pakistan. In total, 446 blood samples were collected from patients and screened for brucellosis using the Rose Bengal Plat Test (RBPT). All the serum samples were investigated for *Brucella* DNA using specific real-time PCR. Age, sex, occupation, urbanicity, socioeconomic status and history of animal contact were recorded and assessed as potential risk factors. The proportion of acute febrile illness patients for whom brucellosis could be suspected was 10.1% by the RBPT. *Brucella* DNA was detected in 26 (5.8%) cases and identified as *B. abortus.* Contact with infected animals, consumption of raw milk and socioeconomic status showed a highly significant (*p* < 0.05) correlation with seropositivity. Elderly patients (19.7% RBPT and 12.1% PCR) and females (13% RBPT and 9.3% PCR) were of high risk of brucellosis. Patients suffering from brucellosis-related manifestations should be screened for brucellosis, especially those in contact with animals or those consuming their unprocessed products, given the increased risk. The results of this study, which highlight that *Brucella abortus* as an important cause of acute febrile illnesses in humans, aid the development of effective control strategies for human brucellosis in Pakistan.

## 1. Introduction

Brucellosis occurs worldwide, especially in the Mediterranean region, the Indian subcontinent, various parts of Africa, and south/central America. It is caused by *Brucella* spp., i.e., *B. abortus* in cattle, *B. melitensis* in sheep and goats, *B. suis* in pigs, and *B. canis* in dogs [1]. Brucellosis is primarily a disease of animals while humans acquire the disease by direct or indirect contact with them [2]. Brucellosis frequently presents as an undifferentiated febrile illness with otherwise varied and non-specific clinical findings [3]. *Brucella* is a facultative intracellular pathogen that has the ability to multiply in phagocytes after entering the body through skin abrasions, inhalation, ingestion or through the conjunctiva [4]. Brucellosis is rarely fatal but causes severe debility and disability in humans. It is a contagious and febrile disease which has a tendency of conversion into chronic illness, becoming a persistent and granulomatous disease [4]. The acute febrile clinical symptoms of brucellosis always overlap with those of other etiological pathogens, and this may lead to misdiagnosis as well as improper antibiotic treatment regimes.

Although several species of *Brucella* can cause human infection, *B. melitensis* and *B. abortus* are the most frequently implicated species [4,5].

The etiology and incidence of acute febrile illness (AFI) represents a major public health problem because clinical diagnosis is usually unreliable and diagnostic tests are often not available in disease endemic areas [6]. Surveillance based on only symptoms results in misdiagnosis because febrile illnesses are caused by clinically indistinguishable pathogens. The accurate diagnosis of febrile illnesses in humans ideally depends on a good surveillance system supported by modern sensitive and specific molecular diagnostic tests [7].

Brucellosis is endemic in many countries and the incidence varies widely from <0.01 to >200 per 100,000 of the population [8,9,10].

The epidemiology of human brucellosis worldwide has drastically changed over the past decade because of various sanitary, socioeconomic, and political reasons, together with the evolution of international travel. Annually, more than 500,000 new human cases of brucellosis are reported worldwide [5,11,12]. In Pakistan, human brucellosis was recorded in 16% of the population and was higher in rural residents and individuals with animal contact [13]. Human brucellosis related to *B. abortus* was reported in 6.9% high-risk professionals such as abattoir workers, veterinarians and farmers from the Potohar plateau of northeastern Pakistan [14].

Brucellosis is a worldwide zoonosis recognized by the Food and Agriculture Organization (FAO), the World Health Organization (WHO) and the World Organization for Animal Health (OIE). In non-endemic areas, human brucellosis is reported in travelers [15]. Most often, human brucellosis is acquired by direct contact with infected animals and their excretions and ingestion of contaminated animal products such as raw milk, raw milk products and undercooked meat [14].

Bovine brucellosis is endemic in Pakistan and *Brucella abortus* has been identified as the causative agent [16,17]. *Brucella abortus* was also found to be the etiological agent of caprine and ovine brucellosis in Pakistan [18].

The isolation of Brucellae from blood, bone marrow, or other tissues is considered as the gold standard for a definite diagnosis of brucellosis. However, cultivation of *Brucella* is time-consuming, hazardous and low-sensitive (70%) when compared with the real-time PCR method (100%) [19,20]. Thus, the diagnosis often relies on indirect evidence of infection. A variety of serological tests have been applied, but at least two serological tests must be combined to avoid false negative results [21].

In developing countries, serological investigations based on rapid slide agglutination tests such as the Rose Bengal Plate Agglutination test (RBPT) are still the mainstay as screening tools for the diagnosis of brucellosis in humans and livestock, but these assays have low specificity [21,22]. The Rose Bengal Plate test does not necessarily assess acute brucellosis, since no assessment of changes in antibody titers is included.

Serodiagnosis is usually made by indirect ELISA due to its high specificity and sensitivity [23]. PCR assays are highly sensitive and specific tools for the rapid diagnosis of human brucellosis and the simultaneous differentiation of *Brucella* species [23]. Recently, brucellosis was found in women who had abortions [17] and *B. abortus* DNA was detected in the sera of high-risk professionals using real-time polymerase chain reaction assays [24]. This study aimed to prove the potential cause and proportion of acute febrile illness that might be due to *Brucella* infection through serological investigations, molecular identification of circulating *Brucella* DNA, and assessment of associated risk factors for brucellosis in patients admitted to Rawalpindi and Islamabad hospitals.

## 2. Materials and Methods

### 2.1. Study Area

The present study was conducted in two hospitals located in Rawalpindi (33.5984° N, 73.0441° E) and in Islamabad (33.7294° N, 73.0931° E), Pakistan. In these main local hospitals, villagers receive treatment for serious diseases. Topographically, the metropolitan area is located on the Potwar plateau of the northeastern part of the country between the Punjab and Azad Kashmir and it is the third largest conurbation in Pakistan.

### 2.2. Sampling and Data Collection

Blood samples were collected from September 2014 to March 2015 from 446 patients (230 males and 216 females) with acute febrile illness (AFI) of an unknown cause admitted to 3 hospitals in Rawalpindi: District Head Quarter Hospital (DHQ), Benazir Bhutto Hospital, (BBH) and Holy Family Hospital Rawalpindi (HFH), and one hospital in Islamabad, the Pakistan Institute of Medical Sciences. Four mL blood was collected aseptically in sterile vacuum bio-tubes and transported to Pir Mehr Ali Shah Arid Agriculture University Rawalpindi (PMAS-UAAR), Pakistan. Serum samples were separated and stored at −20 °C for serological investigation.

Information regarding age, gender, occupation, geographical origin, urbanicity, socioeconomic status, contact with animals and consumption of raw milk was collected on sampling day.

### 2.3. Ethics Approval and Consent to Participate

This study was approved by the Institutional Ethics Committee of the Pir Mehr Ali Shah University of Arid Agriculture Rawalpindi (PMAS-UAAR), Pakistan. The code of Ethical approval was 229 dated 5 August 2015. Oral and written consent was obtained from all participants.

### 2.4. Serological Investigations

In total, 446 sera were primarily tested for the presence of *Brucella* antibodies using RBPT antigen (Veterinary Research Institute, Lahore, Pakistan) [25]. Briefly, 25 µL of antigen preparation was added to 25 µL serum on a glass plate and then mixed gently for 4 min. Agglutination is considered as positive. The positive and negative serum samples used as quality control were kindly provided by the national reference laboratory of brucellosis, Friedrich-Loeffler-Institut, Germany.

### 2.5. DNA Extraction

Genomic DNA was extracted and purified from 446 serum samples using a commercial extraction kit according to the manufacturer’s instructions (Favorgen Biotech Corp, Taiwan, China). The extracted DNAs were stored at −20 °C.

### 2.6. Detection of Brucella DNA

Multiplex real-time PCR for the detection of the *Brucella* genus-specific, *B. abortus*, and *B. melitensis* specific sequences was performed for all seropositive and seronegative samples using target genes *bcsp*31, *alk*B, and BMEI1162, respectively [26]. PCR was carried out using the following primer and probe set (TIB MOLBIOL, Berlin, Germany) (Table 1). The PCR reaction and analysis were performed using a Mx3000P Thermocycler (Stratagene, Agilent Technologies Germany GmbH, Waldbronn, D-76337, Germany). The samples scored positive by the instrument were additionally confirmed by a visual inspection of the graphical plots showing cycle numbers versus fluorescence values [27].

The reference strains of *B. abortus* S-99 (ATCC 23448) and *B. melitensis* 16 M (ATCC 23456) were provided from the national reference laboratory of brucellosis, Friedrich-Loeffler-Institut, Germany, as positive control. The non-*Brucella* Gram negative strain used in the present study to evaluate the specificity of the primers and real-time PCR reaction was *E. coli* (ATCC 10538).

A sample with a fluorescence signal 30 times greater than the mean standard deviation in all wells over cycles 2 through 10 was considered a positive result, whereas a sample yielding a fluorescence signal less than this threshold value was considered negative. Cycle threshold values below 38 cycles were interpreted as positive. The threshold was set automatically by the instrument. The samples scored positive by the instrument were additionally confirmed by visual inspection of the graphical plots showing cycle numbers versus fluorescence values.

### 2.7. Statistical Analysis

The potential risk factors associated with human brucellosis were analyzed using chi-square trend evaluated method M-STAT software (V5.4, Michigan State University, East Lansing 48824, USA). The factors having *p*-values ≤ 5% were considered significant.

## 3. Results

Out of 446 samples, 45 (10.1%) samples were seropositive for brucellosis using RBPT. *Brucella* DNA was detected in 26 (5.8%) serum samples and identified as *B. abortus* by quantitative real-time PCR (Table 2). *Brucella* DNA was detected in seropositive samples while no *Brucella* DNA was amplified from seronegative samples.

The potential risk factors associated with brucellosis are shown in Table 3. Out of 66 patients aged over 40 years, 13 (19.7%) were seropositive for brucellosis (*p* = 0.017). Of patients aged 31 to 40 years, 8.1% were tested positive, as were 10% of patients younger than 30 years.

Female patients showed higher seropositivity (13%) than males (7.4%). Socioeconomic status was identified as a potential risk factor associated with brucellosis as 15.8% poor patients and 9.9% middle-class-income patients were seropositive. The seroprevalence of brucellosis among businessmen, livestock farmers, employers and housewives was 6.9%, 8.6%, 9.1% and 12.6%, respectively. Of In total, 10.7% patients from Rawalpindi and 9.2% patients from Islamabad proved positive. *Brucella* antibodies were detected in 10.8% of patients from rural areas and 9% of patients from urban areas. The patients in direct contact with animals and those who consumed raw milk were found to be at higher risk for brucellosis (*p* = 0.0001 and *p* = 0.0003), respectively.

## 4. Discussion

Brucellosis is a zoonotic disease with public health significance. Acute Febrile Illness (AFI) still represents a common clinical syndrome among patients seeking hospital care. *Brucella* is one of the pathogens causing febrile illness in many developing countries, including Pakistan. The control and eradication of brucellosis is difficult in developing countries such as Pakistan due to the enormous costs related to the surveillance and culling of infected livestock. The disease is endemic in nature and prevails in almost every region of Pakistan. Previous studies have shown the prevalence of brucellosis in humans in Pakistan [14].

Due to the lack of reliable laboratory assistance and tentative clinical management, the diagnosis of acute febrile illnesses in developing countries like Pakistan is still challenging, resulting in inaccurate treatment of patients and routine underreporting of disease [28].

The diagnosis often relies on the indirect evidence of infection [21]. In some countries, serological investigation based on rapid slide agglutination tests, such as the Rose Bengal Plate Agglutination Test (RBPT) using nationally developed antigen, is still the mainstay as a screening tool for the diagnosis of brucellosis in humans and livestock, but these assays have low specificity [21,22,29].

This study was carried out to gain insight on the potential cause of acute febrile illness in patients admitted to different hospitals in the metropolitan areas of Pakistan.

In this study, the patients with acute febrile illness admitted to hospitals do not reflect the general population and it can be assumed that brucellosis may be a prominent reason to seek hospital care, even if the symptoms observed might not be associated with this disease at first. A serious bias might be observed and it is obvious that we cannot generalize our findings. Hence, the number of patients was unexpectedly high. It must be discussed whether patients would profit from a standardized testing for brucellosis in tertiary care hospitals.

In this study, a significant number of patients (10.1%) suffering from acute febrile illness were seropositive for brucellosis. Accordingly, similar findings were detected in high-risk professionals in Pakistan, i.e., 18.5% in abattoir workers and 6.5% in livestock farmers [14]. A similar hospital-based study in a predominantly pastoral community in nearby Kenya indicated a comparable high sero-prevalence among febrile patients of 13.7% [28], highlighting that brucellosis as an important cause of fever. However, the prevalence of probable cases in the current study was higher than that reported among febrile patients in other countries, such as 7% in Egypt (*p* < 0.001) [30] and 7.7% human brucellosis among febrile attendants of urban healthcare facilities in Mali (*p* = 0.03) [31]. Indeed, a strong association has been reported between the prevalence of the disease in animals and in humans. The high prevalence of probable brucellosis among patients consulting for fever in hospital in Uganda ranged from 7% to 14.9% [29], while in Tanzania, it was 0.5% [32]. In acute febrile illness in South Africa, brucellosis with 1.4% (1/74, 95% CI 0.2–9.0%) group-specific total antibodies was also recorded [33]. This variation from our findings can also perhaps be attributed to variations in different serodiagnostic approaches to the disease.

Compared to standard bacteriological methods, PCR is a convenient and safe method for the rapid and accurate diagnosis of human brucellosis in the serum and blood of acute febrile patients [7,23,34,35]. Quantitative real-time PCR, used for the detection of *Brucella* DNA, showed high specificity and sensitivity when compared to conventional PCR, for the detection of the genus as well as of the species of *Brucella* [23]. In the current study, *Brucella* DNA was detected in 5.8 % of patients, which is in agreement with previous studies conducted in Pakistan [24]. In contrast, *Brucella* DNA was detected in 81.9% patients with acute febrile illness (AFI) of an unknown cause in a Northern location in Saudi Arabia [7].

*Brucella abortus* DNA was the only species identified in the investigated blood samples in the present study. It is a common perception that in most of the human brucellosis cases, *B. melitensis* is involved. The most likely reason that *B. abortus* is the only species found in this study is that people in the study area only consume locally produced raw milk from cattle and buffaloes, and *B. abortus* is the only species prevalent in the livestock of this area [16,17]. *Brucella abortus* was previously found to be the causative agent of brucellosis in humans [4]. Of patients with acute febrile illness (AFI) of an unknown cause in a Northern location of Saudi Arabia, *B. abortus* was detected in 10%, *B. melitensis* in 8%, and 82% showed both *B. abortus* and *B. melitensis* [7]. Additionally, another study showed that *B. melitensis* is the leading cause of brucellosis in Saudi Arabia [34] while in Egypt, *B. melitensis* was the only species isolated and recognized as a common cause of AFI [31]. In Tanzania, 7.0% and 15.4% of patients with febrile illnesses participate due to *B. abortus* and *B. melitensis*, respectively, including 6.9% males and 7.2% females [36].

The predominance of brucellosis patients with AFI in this study was recorded in people aged 41 years and older (19.7%). The observed variations across age groups were statistically significant. Similarly, an age-related and increased incidence has been reported in Bangladesh and Lebanon [37,38]. In a previous study in Egypt, the majority of brucellosis patients with AFI were adult males, with approximately one-third citing their principle occupation as farmer [30].

There is no possible reason that explains the higher percentage of AFI in elderly patients in this study.

In this study, *Brucella* antibodies were more highly detected in females (13%) than males (7.4%), although the observed difference was not statistically significant.

Higher, female gender-related seroprevalence has also been reported from neighboring countries such as India and Bangladesh [38,39]. The possible reason for a higher seroprevalence of brucellosis in females is that females in these countries are basically housewives in rural regions and are primarily engaged in the rearing of livestock and the handling of potentially infected products. In contrast to our study, in some countries, males have been found to be more often seropositive, as they predominantly work in livestock farm management in these countries [38,40,41].

Livestock farmers and housewives developed brucellosis more often when compared to employers and businessmen in this study. The highest prevalences were previously reported in livestock farmers in Uganda [42]. A possible reason for these elevated prevalences in livestock farmers and housewives is that these persons are often in contact with animals and animal products, respectively. Elevated brucellosis was detected in humans having contact with animals and animal products in Iran and Turkey [43,44]. A significant association has been reported between the prevalence of the disease in animals and in humans.

In this study, 10.7% of human patients with acute febrile was observed in people of Rawalpindi, and 9.2% in Islamabad. The people in these areas are engaged in the rearing of cattle and buffaloes to satisfy the milk requirements of the twin cities Rawalpindi and Islamabad. These people live in the rural part of Rawalpindi where they are employed in milk production, a profession that increases the chance of acquiring zoonotic diseases like brucellosis. Similarly, elevated brucellosis infection rates were found in different regional areas in Mongolia, Georgia and Jordan [45,46,47].

In this study, the consumption of raw milk was identified as a significant associated risk factor for acute febrile illness caused by *Brucella*. The association of brucellosis in humans and the consumption of raw milk or raw milk products is well documented in rural populations and high-risk professionals from many countries such as Mongolia, Pakistan, Uganda [14,47,48,49].

## 5. Conclusion

This study proves that brucellosis is an underappreciated and often misdiagnosed cause of febrile illness among hospitalized patients in Pakistan.

The present study shows that *B. abortus* is one of the important etiological agents of AFI in elderly patients in the study area of the Punjab region.

This study has underscored the importance of febrile bacterial diseases, including zoonoses such as brucellosis in febrile patients, which should be considered by clinicians in differential diagnoses of other febrile diseases. This would allow febrile patients to receive the correct diagnosis and the facilitation of accurate and prompt treatment. Additional research regarding local risk factors for human brucellosis and closer collaboration between human and animal health experts are paramount to developing evidence-based prevention strategies in Pakistan.

## Figures and Tables

**Table 1:** Primer and probe sequences used for the detection of *Brucella* genus and species.

Target	Primer/Probe	Target Gene	Sequence
*Brucella* genus	Forward	bcsp31	5′-GCTCGGTTGCCAATATCAATGC-3′
	Reverse		5′-GGGTAAAGCGTCGCCAGAAG-3′
	Probe		FAM-AAATCTTCCACCTTGCCCTTGCCATCA-BHQ1′
*B. abortus*	Forward	IS711	5′-GCGGCTTTTCTATCACGGTATTC-3′
	Reverse		5′-CATGCGCTATGATCTGGTTACG-3′
	Probe		FAM-CGCTCATGCTCGCCAGACTTCAATG-BHQ1
*B. melitensis*	Forward	IS711	5′-AACAAGCGGCACCCCTAAAA-3′
	Reverse		5′-CATGCGCTATGATCTGGTTACG-3′
	Probe		FAM-CAGGAGTGTTTCGGCTCAGAATAATCCACA-BHQ1

**Table 2:** Comparison of the results of different serological and molecular methods used to investigate the prevalence of human brucellosis in the Rawalpindi/Islamabad region, Pakistan.

Method Used		Sera Positive/Total No.	Prevalence
Conventional screening test	RBPT	45/446	10.1%
Molecular detection	Real time-PCR	26/446	5.8%

**Table 3:** Detection of anti-*Brucella* antibodies and DNA in AFI patients using RBPT and real-time PCR, respectively, in three tertiary care hospitals of the Rawalpindi/Islamabad region, Pakistan, according to their potential risk factors.

Risk Factors	Categories	Number of Patients	Seropositive (%)	DNA (%)	*p*-Value
Age (Years)	21 to 30	60	6 (10%)	4 (6.6%)	0.017
	31 to 40	320	26 (8.1%)	14 (4.4%)	
	41 and above	66	13 (19.7%)	8 (12.1%)	
Gender	Male	230	17 (7.4%)	6 (2.6%)	0.07
	Female	216	28 (13%)	20 (9.3%)	
Occupation	Livestock Farmers	116	10 (8.6%)	6 (5.2%)	0.4
	Businessmen	102	7 (6.9%)	3 (3.0%)	
	Employer	22	2 (9.1%)	0	
	Housewives	206	26 (12.6%)	17 (8.3%)	
Geographical region	Rawalpindi	261	28 (10.7%)	16 (6.1%)	0.619
	Islamabad	185	17 (9.2%)	10 (5.4%)	
Urbanicity	Rural	268	29 (10.8%)	18 (6.7%)	0.639
	Urban	178	16 (9%)	8 (4.5%)	
Socioeconomic status	Middle	427	42 (9.8%)	18 (4.2%)	0.56
	Poor	19	3 (15.8%)	1 (5.3%)	
Contact with animals	Yes	200	33 (16.5%)	18 (9.0%)	0.0001
	No	246	12 (4.9%)	8 (3.3%)	
Consumption of raw milk	Yes	230	30 (13%)	19 (8.3%)	0.0003
	No	216	15 (6.9%)	7 (3.2%)	
